# Preventive Effects of Three Polysaccharides on the Oxidative Stress Induced by Acrylamide in a *Saccharomyces cerevisiae* Model

**DOI:** 10.3390/md18080395

**Published:** 2020-07-28

**Authors:** Zhen Lin, Yu Zhang, Fangping Li, Xiaohui Tan, Ping Luo, Huazhong Liu

**Affiliations:** College of Chemistry & Environmental Science, Guangdong Ocean University, Zhanjiang 524088, China; linz199771@163.com (Z.L.); joanne96zy@163.com (Y.Z.); 15709482571@163.com (F.L.); tanxiaohuiii@163.com (X.T.); luopingna@163.com (P.L.)

**Keywords:** acrylamide, polysaccharides from *Sepia esculenta* ink, fucoidan from *Laminaria japonica*, polysaccharides from *Eleocharistuberosa* peel, oxidative stress, *Saccharomyces cerevisiae*

## Abstract

*Saccharomyces cerevisiae* was used as a model to explore the preventive effect of two marine polysaccharides separately derived from *Sepia esculenta* ink (SIP) and *Laminaria japonica* (FL) as well as one terrestrial polysaccharides from *Eleocharis tuberosa* peel (WCPP) on toxic injury induced by acrylamide (AA). The growth of yeast was evaluated by kinetics indexes including doubling time, lag phase and maximum proliferation density. Meanwhile, intracellular redox state was determined by contents of MDA and GSH, and SOD activity. The results showed that AA inhibited yeast growth and destroyed the antioxidant defense system. Supplement with polysaccharides, the oxidative damage of cells was alleviated. According to the growth recovery of yeast, FL and WCPP had similar degree of capacity against AA associated cytotoxicity, while SIP was 1.5~2 folds as strong as FL and WCPP. SIP and FL significantly reduced production of MDA by AA administration. Moreover, SIP, FL and WCPP increased SOD activity and repressed GSH depletion caused by AA.

## 1. Introduction

Acrylamide (AA) is a monomeric chemical that can be used as polymeric flocculant in textile and paper industry after forming polyacrylamide [1]. In 1994, AA was classified as group 2A carcinogen by the International Agency for Research on Cancer [2]. Due to the Maillard reaction between asparagine residues and reducing sugars, heat-processed foodstuffs, such as potato products, bread, cereals and coffee, produce AA that is one of the main environmental sources of human exposure [3,4]. One of the important toxicological mechanisms is that AA mediates intracellular oxidative stress by promoting generation of reactive oxygen species (ROS), leading to the depletion of glutathione (GSH), and consequent DNA damage [5,6]. In recent years, maintaining the redox equilibrium in tissues or cells with natural or synthetic active compounds has become a research hotspot, which was considered as a potential treatment strategy for diseases caused by oxidative stress [7,8,9]. Among these active compounds, natural polysaccharides have a wide range of beneficially therapeutic effects and health-promoting properties. As antioxidants in vivo and in vitro, polysaccharides can effectively scavenge free radicals and protect organisms from oxidative damage [10]. 

Squid ink polysaccharide (SIP) is a type of natural bioactive substance isolated from cuttlefish ink, and has been proven to have multifunctional properties [11,12,13,14]. In our previous study, it was revealed that SIP could ameliorate the oxidative stress damage caused by cyclophosphamide (CP) though activating Nrf2/ARE (Nuclear factor-erythroid 2-related factor 2/Antioxidant response element) pathway to up-regulate the expression of antioxidases and Ⅱ phase enzyme genes [13,14]. In addition, the activity of SIP preventing chemotherapeutic damage was also reflected in inhibiting the programmed cell death caused by CP and its metabolite acrolein (ACR) [11,12]. Fucoidan is also a marine-derived polysaccharide isolated from brown seaweeds such as *Laminaria japonica*, *Fucus vesiculosus* and *Sargassum cinereum*. This kind of polysaccharide is reported to possess potentially diverse medicinal values, such as antioxidant, anti-inflammatory and renoprotective activities, etc. The protective effect on the kidney has made progress in clinical research which was demonstrated by clinical treatment for renal diseases for nearly 20 years [15]. In terms of antioxidants, our previous study found that the *Laminaria japonica*-derived fucoidan (FL), same as allopurinol, restored the content of malondialdehyde (MDA) in the liver of adenine-induced hyperuricemia mice to normal [16]. Meanwhile, FL also restored activities of both superoxide dismutase (SOD) and catalase (CAT) in the liver to normal status, which allopurinol failed to [16]. Chinese water chestnut (*Eleocharis dulcis*), one of the favorite foods in Asia due to its unique taste, can be used as a folk medicine to treat hypertension, chronic nephritis, constipation, and pharyngitis [17]. The peel that is discarded as a by-product during the processing of Chinese water chestnut, constitutes approximately 20% (w/w) of the whole fruit, so the recycle of the Chinese water chestnut peel (CWCP) is beneficial to the environment and economy [17]. Previous studies on the activity of CWCP have shown that its flavonoids have antioxidant, anticancer, antibacterial and nitrite scavenging functions [17,18]. However, research about the extraction and activity of polysaccharides in CWCP (WCPP) has not been reported internationally.

In order to explore whether the antioxidant activity of polysaccharides can alleviate the oxidative stress induced by AA, yeast *S. cerevisiae*, a well-consolidated eukaryotic model in toxicological studies, was used as the oxidative damage model in this study. It was found that SIP, FL and WCPP can reduce the damage caused by AA to some extent by regulating the antioxidant system (including SOD, GSH and MDA) of yeast.

## 2. Results

### 2.1. Cytotoxicity of Acrylamide to S. Cerevisiae

**Figure 1:**
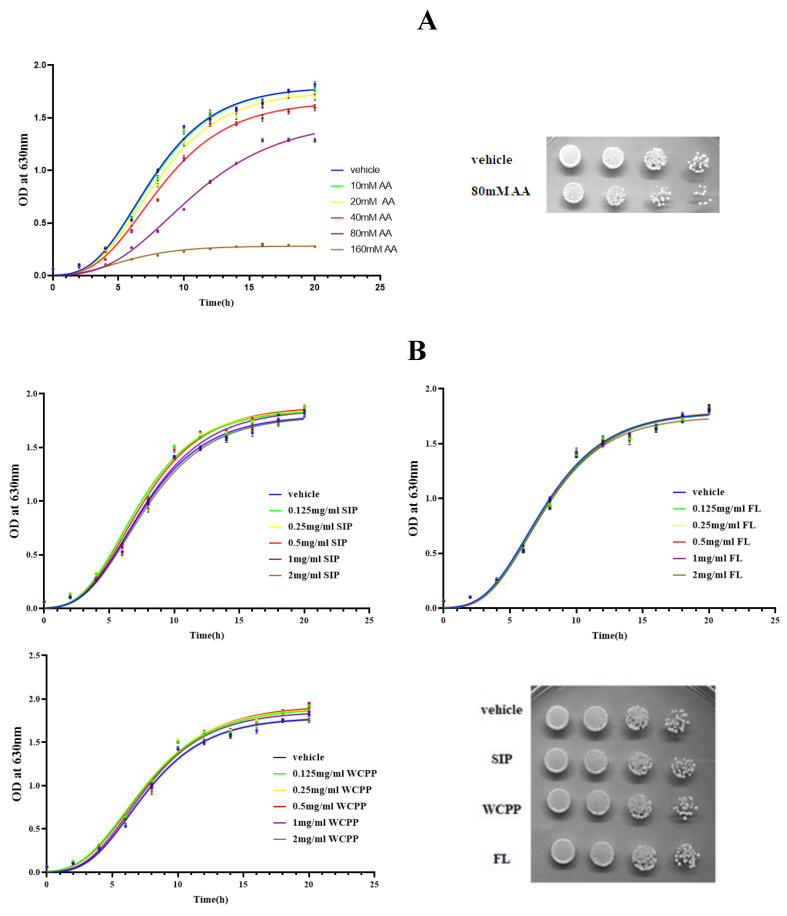
(**A**) Growth curve of yeast under AA administration at concentrations of 10, 20, 40, 80 and 160 mM and the growth of yeast after cultured 6 h in the presence of AA at 80 mM using spot assay. (**B**) Growth curve of yeast under SIP or FL or WCPP administration at concentrations of 0.125, 0.25, 0.5, 1 and 2 mg/mL and the growth of yeast after cultured 6 h in the presence of SIP or FL or WCPP at 0.5 mg/mL using spot assay.

Compared with the vehicle group, the number of cells decreased and the rate of growth was delayed under AA treatment (Figure 1A). Data in Table 1 showed that this cytotoxicity in a dose-dependent manner, resulting in an IC50 value of 92.64 ± 3.30 mM. From 40 mM onwards, the proliferation of cell was significantly inhibited by AA and the number of cells at each time point was significantly less than that of the vehicle group (*p* < 0.05). In the spot assay (Figure 1A), the formation of yeast colonies after AA treatment of 80 mM was less than that of the vehicle group, which also reflected the toxicity of AA to yeast cells. From the growth dynamics perspective, the doubling time of cell proliferation in the presence of 20 mM, 40 mM, 80 mM and 160 mM acrylamide (Table 2) was significantly prolonged by 4%, 10.6%, 43.0% and 149.7% (*p* < 0.05). In addition, under 20 mM, 40 mM, 80 mM and 160 mM AA administration, the lag phase (1/K) of cell growth was significantly prolonged by 4.8%, 9.9%, 43.7% and 68.7% (*p* < 0.05). The maximum density of cell proliferation (Ym) was also restricted, in which cell treated with 40 mM and 80 mMAA decreased significantly by 7.8% and 17.2% (*p* < 0.05). When the AA concentration was 160 mM, Ym was only 17.2% that of the vehicle group (Table 3). According to IC50 value, AA with a concentration of 80 mM was selected for the next damage experiment.

### 2.2. The Benefit Effect of SIP and WCPP on the Growth of Yeast under Natural Conditions

The growth curves (Figure 1B) and corresponding OD_630_ values in the presence of three kinds of polysaccharides revealed that treated cell growth was promoted by SIP and WCPP ( S1) at low concentration (including 0.125, 0.25, 0.5 mg/mL) (*p* < 0.05). However, within the concentration range of polysaccharide (0.125~2 mg/mL), FL was nonsignificant effect on cell growth (*p* > 0.05). In the spot assay, polysaccharides had no significant effect on the formation of yeast colonies

In terms of multiplication rate, Table 2 showed that SIP at concentrations of 0.125, 0.25 and 0.5 mg/mL reduced the doubling time (*p* < 0.05). Overall, 0.25 mg/mL of SIP administration had the strongest promoting effect, which is 5.3% shorter than the vehicle group. Likewise, in the presence of 0.125, 0.25, 0.5, and 1 mg/mL WCPP, the doubling time was also reduced (*p* < 0.05), and in the presence of 0.25 mg/mL WCPP, the doubling time was reduced the most, with a reduction of 5.1% compared with untreated yeast. As for FL, there was no significant effect on the multiplication rate of yeast (*p* > 0.05).

Through the comparison of growth kinetic parameters, the activities of SIP and WCPP in promoting proliferation can also be confirmed. Both SIP and WCPP at concentrations of 0.125 and 0.25 mg/mL were able to shorten the lag time of yeast inoculation in the new environment (*p* < 0.05) (Table 3). It is worth noting that when the concentration of SIP reached 2 mg/mL, its proliferative activity turned to inhibition, that is, the doubling time was prolonged by 2.1% compared with the vehicle group. Moreover, beside 2 mg/mL, the maximum proliferation density of the SIP and WCPP groups was higher than that of the vehicle group (*p* < 0.05).

### 2.3. Prevention of SIP, FL and WCPP on AA-Mediated Cytotoxicity

**Figure 2:**
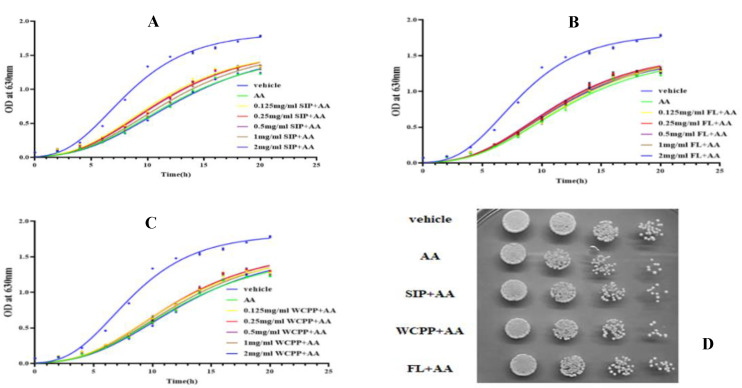
(**A**–**C**) Growth curve of yeast under 80 mM AA co-treated with SIP or FL or WCPP administration at concentrations of 0.125, 0.25, 0.5, 1 and 2 mg/mL. (**D**) The growth of yeast after cultured 6 h in the presence of 80 mM AA combined with SIP or FL or WCPP at 0.5 mg/mL using spot assay. (**C**,**D**).

In order to evaluate whether the antioxidant activity of the three polysaccharides could alleviate the inhibitory effect of AA on yeast, a series of polysaccharides with a concentration gradient and AA of 80 mM were combined to intervene the cells. The results (Figure 2A) showed that in the combination of SIP (0.125~1 mg/mL), FL (0.5~1 mg/mL), or WCPP (0.5~1 mg/mL) and AA, the number of cells at almost all time points was higher than that of AA treated alone (*p*< 0.05). Accordingly, the inhibition rates of AA on cells were also reduced in the presence of polysaccharides. The inhibition rates of the SIP (0.125~1 mg/mL) + 80 mM AA group were reduced by 25.3%, 21.3%, 19.7% and 9.8% compared with the 80 mM AA group (*p* < 0.05), while inhibition rates of the FL (0.25~2 mg/mL) + 80 mM AA group were reduced by 12.8%, 14.2%, 9.1% and 6.8% (*p* < 0.05), as for inhibition rates of the WCPP (0.125~1 mg/mL) + 80 mM AA group were reduced by 12.1%, 14.1%, 13.5% and 6.2% (*p* < 0.05; Table 4). In addition, 0.5 mg/mL of the three polysaccharides also weakened the inhibitory effect of AA on colony formation in spot assay (Figure 2B).

The yeast proliferation rate was slowed down due to the inhibitory effect of AA toxicity on cells, and this effect could be alleviated by polysaccharides. Data (Table 4) showed that the concentrations of 0.125, 0.25, 0.5 and 1 mg/mL of SIP exhibited the significant inhibitory effect against AA-induced slowing of cell growth (*p* < 0.05), and the cell doubling time was reduced by 14.6%, 12.6%, 11.5% and 5.6%, respectively, compared with that without SIP (*p* < 0.05). Similar effect was observed when AA was combined with FL or WCPP. Cell exposure to AA and supplemented with FL restored the doubling time by 6.5%, 7.4%, 4.1% and 2.5% at 0.25, 0.5, 1 and 2 mg/mL (*p* < 0.05). In the presence of WCPP (0.125~1 mg/mL), the AA inhibition of the doubling time was reduced by 8.5%, 8.3%, 6.5% and 1.3% (*p* < 0.05). However, when the concentration of WCPP reached 2 mg/mL, the cell doubling time was longer than when only AA was present, which indicated these two reagents combine to inhibit the cell proliferation rate (*p* < 0.05).

The presence of polysaccharides reduced the environmental pressure on the growth of cells in a medium containing AA, as demonstrated by kinetic parameters (Table 5). From the perspective of the lag time, the SIP of 0.125, 0.25, 0.5 and 1 mg/mL can shorten this index in the presence of AA by 11.2%, 8.6%, 6.9%, 2.1% (*p* < 0.05). FL at concentrations of 0.25, 0.5, 1 and 2 mg/mL showed antagonistic effects on AA prolonging the cell lag time, which resulted in a reduction of 5.0%, 6.5%, 3.6%, and 4.1% (*p* < 0.05). The concentration of 0.125, 0.25 and 0.5 mg/mL of WCPP reduced the lag time of AA treated yeast by 5.1%, 3.8% and 2.7% (*p* < 0.05). When the concentration was at the maximum value of the range set, SIP and WCPP did not exhibit a protective effect, on the contrary, they intensified the inhibition of AA on the lag time of yeast. Beyond that, SIP and WCPP also alleviated the maximum proliferation density restriction of yeast under AA environment. 

In order to explore the effect of polysaccharides on intracellular redox balance when used as cell protectants, three polysaccharides of 0.5 mg/mL and AA of 80 mM were selected to synergistically intervening yeast growth in following experiment.

### 2.4. Attenuation of SIP, FL and WCPP on the Increase in Lipid Peroxidation Caused by AA

**Figure 3:**
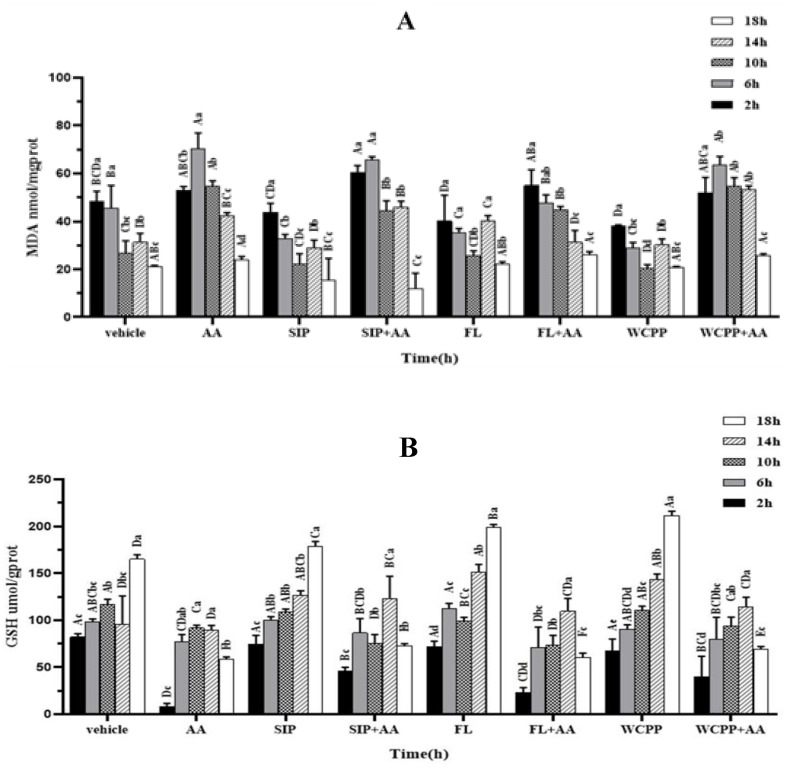

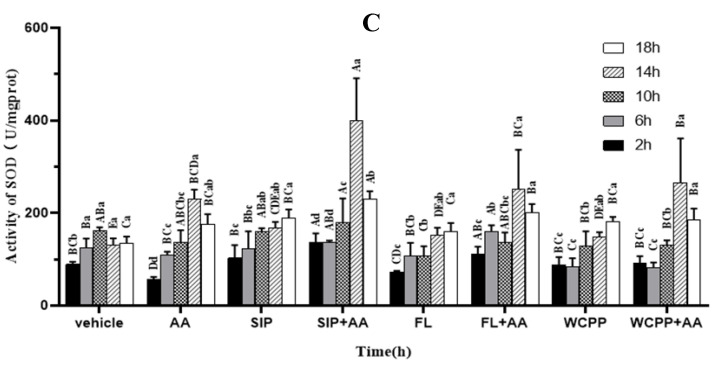
(**A**–**C**) The trend of MDA level, GSH level and SOD activity at 2, 6, 10, 14 and 18 h in each group. ABCD indicates *p* < 0.05 between groups, abcd indicates *p* < 0.05 within group.

A two-way repeated measures analysis of variance indicated a significant interaction between different time points and different treatments on MDA level in cells [F (28, 64) = 5.10, *p* < 0.001]. From the perspective of the main effect of time, MDA level decreased gradually with time [F (4, 13) = 111.56, *p* < 0.001], but this change presented inconsistency among different groups. The trend of MDA level within each group showed that the maximum value of MDA level in the groups without AA appeared at the first 2 h, and gradually decreased with the extension of culture time (Figure 3A). Among them, MDA levels in the SIP group and WCPP group decreased significantly from 2 to 6 h (*p* < 0.05), while those in other groups decreased gently. As for the AA group, MDA content increased first and then decreased, among which, MDA content increased significantly by 32.6% from 2 to 6 h (*p* < 0.05). However, this increase became insignificant after the participation of SIP, and MDA content even showed a trend of decreasing rather than increasing after the participation of FL.

Figure 3A also shows the simple effect of analyzing the MDA content between different groups, which revealed that at 2 h, there was no significant difference in MDA levels in the AA and the three polysaccharide groups compared with the normal cells (*p* < 0.05). When the cells were cultured for 6 h, the MDA level in the AA group increased significantly by 54.2% compared with the vehicle group, while co-treatment with FL obviously decreased AA-induced MDA level by 32.0% (*p* < 0.05). Moreover, SIP, FL and WCPP treatment alone, respectively, caused a significantly down-regulation (8.9%, 21.3% and 16.8%) of MDA levels in yeast, compared with the vehicle group at 6 h (*p* < 0.05). At 10 h, the MDA level of the AA group was higher than that of the normal group by 50.7% (*p* < 0.05). At this time, SIP also showed an antagonistic effect on the increase in AA-induced MDA level, which resulted in an 18.6% decreasing (*p* < 0.05). At 14 h, the MDA level of cells treated with AA was still higher than that of normal cells, and the intervention of FL significantly reduced this value (*p* < 0.05). At the last time point, the MDA level of each group reached the lowest, and the MDA level of the SIP + AA group was significantly lower than that of the other groups (*p* < 0.05).

### 2.5. Antagonistic Effects of SIP and WCPP on Intracellular GSH Depletion Induced by AA

As an important intracellular reductant, the content of GSH was also monitored in this study. The results of two-way repeated measures analysis of variance revealed a significant “time × group” interaction [F (28, 64) = 4.08, *p* < 0.001]. The GSH level in different groups showed the following two different trends with the change of culture time (Figure 3B). In the groups without AA, the GSH content gradually increased, especially between 14 h and 18 h (*p* < 0.05). While in the groups with AA, the GSH content first increased and then decreased, which was manifested in a significant increase between 2 and 6 h, and a significant decrease between 14 and 18 h (*p* < 0.05).

A comparison of simple effects between the groups revealed an obvious loss of GSH in AA-treated cells of 90.5% versus vehicle group two hours after inoculation (*p* < 0.05). The depletion of GSH was significantly restored to 55% and 48.5% of normal levels by the addition of SIP and WCPP, respectively (*p* < 0.05). When cultured for 6 h, the GSH level of cells in the AA group rose to no significant difference from that in the normal group (*p* > 0.05). It is also worth noting that GSH levels in the cells treated by SIP, FL and WCPP alone were significantly higher than those in the vehicle group at 14 h and 18 h (*p* < 0.05).

### 2.6. Prevention of SIP, FL and WCPP on AA Induced SOD Activity Disruption

The effect of time and group interaction on SOD activity in cells was also statistically significant (F (28, 64) = 3.21, *p* < 0.001). From a within-groups perspective, SOD activity increased significantly from 2 h to 6 h, and then leveled off from 6 h to 18 h in normal cell (*p* < 0.05, Figure 3C). In the SIP, FL and WCPP groups, SOD activity showed an increasing trend, and the activity was most potent at 18 h. As for the AA group and combination group, it exhibited a gradually increasing trend from 2 to 14 h, in particular, a significant sudden jump appeared from 10 to 14h and then decreased from 14 to 18 h (*p* < 0.05).

As shown in Figure 3C, yeast was inoculated into the medium containing AA for two hours, SOD activity was significantly lower than that of the group without AA stress (*p* < 0.05). At the time of 2 h, with the participation of SIP, FL and WCPP, the inhibitory effect of AA on SOD activity was antagonized, in which FL and WCPP restored SOD activity to the same level as normal cells, and SIP even significantly increased SOD activity by 52.5% compared with normal group (*p* < 0.05). Similarly, at 6 h, the SOD activity of the AA supplemented with FL group was significantly higher than the normal level (*p* < 0.05). At 14 h, the SOD activity of AA group was distinctly higher than that of the vehicle group, and that value of AA co-treated with FL or WCPP group was also at the same level (*p* < 0.05). Interestingly, in the AA group supplemented with SIP, the SOD activity was further significantly increased than that of AA group by 73.9% (*p* < 0.05). At the last observation time point, SOD activity in the AA group was no different from that in the normal cell, while in the AA groups combined with polysaccharide, SOD activity was still significantly higher than that in the normal cells (*p* < 0.05). 

## 3. Discussion

Since it is recognized that AA is widely derived from the diet during high temperature processing, the harmful effect of AA on human health has also been well-studied [19]. In particular, AA has been demonstrated to be neurotoxic in humans and laboratory animals [20,21,22], resulting in sensory and motor dysfunction, skeletal muscle weakness, weight loss, and ataxia [23]. One of the important mechanisms by which AA induces neurotoxicity is that it can destroy the redox balance and induce oxidative stress in neurons [24,25]. Under oxidative stress of AA, intracellular ROS were greatly produced, GSH levels decreased rapidly, and a series of oxidative stress followed [26]. In response to oxidative stress, Nrf2 pathway, which is closely related to anti-oxidation, is rapidly activated in cells to counteract the negative effects of oxidative stress [26]. However, if excessive accumulation of ROS cannot be reversed, oxidative stress will lead to cell apoptosis [27]. 

In order to antagonize AA-induced oxidative stress in cells, recent studies have focused on finding active substances that can alleviate AA cytotoxicity. Various active substances, such as cerium oxide, quercetin, ellagic acid, L-carnitine and diosmin, have been found to be potential cell protectants [28,29,30,31,32]. Especially, natural antioxidant active substances have attracted much attention due to their high antioxidant capacity, low toxicity and few side effects [33]. Polysaccharide is also a natural antioxidant widely existing in nature, and it is rare to study its application in protecting cells from AA oxidative stress. Therefore, in this study, yeast was used as a model to explore the protective effect of polysaccharides from three different sources on cells exposed to AA, so as to provide a preliminary basis for expanding the application of polysaccharides in food processing.

The toxicity test showed that the yeast exposed to AA was significantly inhibited in a dose-dependent manner, with an IC50 value of 92.64 ± 3.30 mM. The inhibition of AA on yeast proliferation was mainly reflected in three aspects: firstly, it reduced the rate of yeast proliferation; secondly, the yeast inoculated in the medium containing AA had a longer lag phase; thirdly, the maximum density of yeast proliferation was significantly reduced. This result is consistent with the study on AA cytotoxicity using yeast as a model, but previous studies did not explore the effect of AA on the growth kinetics parameters of yeast [3,4].

On the contrary, in the presence of SIP and WCPP, the growth of yeast was promoted, which was characterized by increased growth rate, shortened lag phase and elevated maximum proliferation density. Meanwhile, the degree of growth promotion of yeast was similar between these two polysaccharides. However, FL had no significant effect on the growth of normal yeast. It should be noted that the growth-promoting effect of SIP and WCPP were only present at low concentrations, but not at high concentrations, and even SIP showed an inhibitory effect on yeast growth at 2 mg/mL. This inhibition is derived from the antimicrobial effect of polysaccharides, and numerous studies have demonstrated that polysaccharides, especially those rich in sulfuric acid and low molecular weight, can inhibit the growth of a variety of microorganisms [34].

Under the protection of polysaccharides, the growth arrest effect of AA on yeast was obviously alleviated. This protective effect can be proved from the fact that after SIP, FL and WCPP were supplemented respectively, the inhibition rate of AA on cell growth was reduced, the delaying effect on growth rate was decreased, the effect on prolonging the lag time was weakened, and the restriction on maximum proliferation density was alleviated. These three polysaccharides antagonize the negative effects of AA on yeast growth to different degrees. Considering the recovery effect of polysaccharides on various growth indicators of yeast under AA induced oxidative stress, SIP and WCPP had the best protective effect at 0.125 mg/mL, while FL had the best protective effect at 0.5 mg/mL. At the optimal concentration, FL and WCPP had similar levels of protection, while the antagonistic effect of SIP on oxidative stress was 1.5~2 folds than that of FL and WCPP. It is worth emphasizing that this protective effect is not dose-dependent and that the protective effect of polysaccharides is more significant at lower concentrations, which is similar to the promoting effect of polysaccharides on yeast growth when used alone.

In the presence of AA, the production of MDA, the index of lipid peroxidation, in yeast cells increased, which was consistent with the results of other experimental models [28,31]. It was considered as a sign of cell damage after AA destroyed the oxidant/antioxidant balance in cells [31]. The trend of MDA level in yeast cells exposed to AA was significantly different from that of normal cells. At the time point of the first observation, there was no difference in MDA levels between the AA group and the vehicle group. With the culture time up to 6 h, content of MDA in normal cells decreased, while MDA level in AA group increased, which was significantly higher than that in normal cells at the same time. Yeast possesses an antioxidant defense to antagonize the cytotoxicity to a certain extent [35]. Hence, in the lag phase, intracellular basic antioxidant system resisted the AA induced oxidative stress, resulting in the same MDA level as normal cells. However, this self-derived antagonistic effect could not be sustained all the time. As the duration on AA associated stress was prolonged, the MDA production in cells would increase significantly. Interestingly, at 6 h, MDA levels in the AA + FL group were not significantly different from those in the normal cell, suggesting that FL as a cell protectant greatly reduced the formation of lipid peroxides in yeast under AA mediated oxidative stress. The results were similar to FL restored MDA levels in the liver of adenine-induced hyperuricemia mice to normal value [16]. Although, the antagonistic effect of SIP and WCPP on AA induced MDA was not significant at 6 h, compared with cells under natural conditions, the presence of SIP and WCPP reduces MDA. FL treated alone also reduced MDA production at 6 and 10 h, which may be one reason of these three polysaccharides promote the natural growth of yeast by reducing lipid peroxidation during natural cell growth. Cells exposed to AA for more than 6 h were observed MDA decreased gradually, which may be due to the activation of the intracellular antioxidant defense system. However, the value continued to be higher than normal cells at 10 h and 14 h, and it was not until 18 h later that MDA production was at the same level between the AA group and the vehicle group. From 10 h to 18 h, MDA levels also exhibit a decreasing trend in the groups treated with AA supplement with three different polysaccharides, respectively. Compared with the yeast exposed to AA, the generation of MDA was reduced in the SIP + AA group at 10 h and 18 h, and in the FL + AA group at 10 h and 14 h. As for WCPP, it has no significant influence on the trend of MDA caused by AA. 

The biotransformation of ACR is carried out by binding with GSH, a non-enzymatic antioxidant reducing agent, and decarboxylation, and then it is metabolized to glycidamide (GA) by cytochrome P4502E1 (CYP2E1) [36]. Hence, depletion of GSH is commonly seen in various reports on AA induced oxidative stress [26,28,32]. In this study, the GSH in the AA group was only one-tenth of the normal cells at 2 h, which indicated that AA consumes a lot of GSH from the cell. This also explains that at 2 h, the lipid peroxides in the AA group were not different from those in the normal group, which could be attributed to the defense effect of GSH. However, in the AA + SIP and AA + WCPP groups, the depletion of GSH was significantly reduced. At 6 h, GSH in the AA group was up-regulated compared with the previous time point, and at the same level as the vehicle group. The up-regulation of intracellular GSH depends on the regulation of Nrf2 to γ-glutamyl cysteine synthetase the rate-limiting enzymes of GSH biosynthesis [37]. Previous studies have confirmed that SIP and fucoidan can activate Nrf2 and mediate GSH level rise in chemo-drug mediated oxidative stress [14,38] From 10 h to 18 h, the GSH level of normal cells gradually increased, while the GSH level of cells in the AA group gradually decreased, which may be due to AA damaged the cell genes. AA is metabolized to GA and binds to DNA and proteins more actively, leading to glycidamide-DNA adduct formation, which could be participated in ACR mutagenicity [36]. Under the protection of polysaccharides, the trend of GSH was the same as that of cells treated with AA alone, indicating that polysaccharides could effectively protect cells exposed to AA at an early stage, but did not completely reverse the genetic damage caused by long-term oxidative stress. GSH in the polysaccharide monotherapy groups were no different from that in the vehicle group from 2 h to 14 h. At 18 h, the GSH of polysaccharide group were higher than that of normal cells, which indicated that polysaccharides also had an up-regulation effect on the GSH of non-oxidative stress cells, but the degree and time point were different from the elevation of GSH by polysaccharides in oxidative stress cells.

SOD, a metalloenzyme belong to antioxidant enzymes, detoxify oxidative stress through transforms the superoxide radical into hydrogen peroxide which was then degraded into water and oxygen by catalase and glutathione peroxidase [24,39]. The effect of AA on SOD activity in different experimental models has been reported in many previous studies, but the results seem to be inconsistent. The observation result of some studies is that under the administration of AA, SOD enzyme activity was significantly inhibited, which indicates that AA induced redox imbalance and the antioxidant defense system failed to combat the influx of ROS [31,40,41,42]. In contrast, another part of the study showed that the stimulation of AA increased the activity of SOD, and it is thought to be a compensatory mechanism against oxidative stress [3,20,43]. This inconsistency may be attributed to the following three aspects: different experimental models, different doses of AA, and different observation time points. In this study, SOD activity in the cells exposed to AA for 2 h was lower than that in the vehicle group, and the results obtained by using a lower dose of AA (about 14 mM) in similar studies were that SOD was activated [3]. Therefore, under the intervention of high concentration of AA, SOD activity will be inhibited. However, in the medium supplemented with polysaccharides, the polysaccharides played a protective role and SOD activity was not inhibited. Among them, the SOD activity of the SIP + AA group was even higher than that of the vehicle group, indicating that SIP significantly activated the SOD activity of the cells under AA-induced oxidative stress. From 2 h to 14 h, SOD activity of AA group was gradually enhanced, and reached the strongest at 14 h, which was significantly higher than that of the vehicle group. This confirmed that SOD activity under oxidative stress of AA observed at different time points would yield different results compared with that of the normal group. The SOD activity of the group using AA and polysaccharide in combination also showed a similar trend. Significantly, the SOD activity of the SIP + AA group was higher than that of the AA group at 14 h. At 18 h, SOD activity of these groups decreased relative to the previous time point, while the trend of SOD activity in the vehicle group was relatively stable. As for the data of the polysaccharide treatment alone showed that the polysaccharide had no obvious effect on SOD activity in the naturally growing cells.

Although the three macromolecules investigated in this study are polysaccharides, they come from different species and are structurally quite different from each other. In our previous study, marine polysaccharide SIP was rich in galactosamine (GalN) and arabinose (Ara) residues [12]. FL, another marine polysaccharide, is dominated by fucose (Fuc) residues [16]. The difference in molecular composition leads to the difference in antioxidant capacity, but how the two molecular structures determine biological activity is unknown and worthy of further investigation.

As a non-pathogenic and relatively simple unicellular organism, *S. cerevisiae* is an ideal model for functional toxicology research due to its rapid proliferation, robust environmental adaptability, high degree of functional conservatism with complex eukaryotes, and the fact that almost every gene has functional information [44]. The protective effect of polysaccharides was rapidly and conveniently evaluated through comparing the growth kinetics and antioxidant biomarkers of yeast in different groups. Moreover, this model also allows the antioxidant capacity of polysaccharides in response to specific oxidants to be more clearly demonstrated. Traditionally, some vitro antioxidant experiments such as trolox equivalence antioxidant capacity (TEAC) assay, ferric ion reducing antioxidant power (FRAP) assay, and 2,2-diphenyl-1-picrylhydrazyl radical (DPPH) scavenging can be used to evaluate the antioxidant capacity of polysaccharides. However, this is full of limitations, Granato et al. points out that it is difficult to fully evaluate the antioxidant effect of food biomaterials solely based on in vitro antioxidant experiments, and the results are likely to be contradictory [45]. Compared with in vitro antioxidant experiments, it is more convincing to evaluate the antioxidant capacity of polysaccharides by cell model, because the antioxidant capacity of polysaccharides is not only reflected in the scavenging of free radicals, but also plays a regulatory role in the signaling pathways and enzymes related to antioxidant in cells [46].

In conclusion, AA mediates dose-dependent toxicity to yeast via inducing oxidative stress, which was demonstrated by prolonged doubling time and lag phase, declined maximum cell proliferation density and disrupted antioxidant capacity. However, administration of natural polysaccharides, such as SIP, WCPP and FL, ameliorated the toxic effect AA induced in yeast; moreover, SIP has more effective capacity than WCPP and FL. The data re-confirm that biological polysaccharides are natural antioxidants that can be used to attenuate oxidant-induced intracellular oxidative damage, and also report that SIP has high-efficient in vivo antioxidant capacity on the basis of a eukaryotic microorganism model, although the in vivo antioxidant property of SIP has been reported many times by our previous findings in mice. This work also demonstrates that SIP has stronger in vivo antioxidant capacity than other polysaccharides, such as FL and WCPP. Based on the comprehensive results, it can be concluded not only that SIP is more suitable for developing into natural antioxidants than FL and WCPP, maybe including other polysaccharides, and also that SIP may be more suitable for developing into clinical drugs or adjuvant agents than FL that has been used clinically for nearly 20 years.

## 4. Materials and Methods

### 4.1. Preparation of SIP

Based on the method of our laboratory [11], the extraction and separation process is slightly adjusted. Fresh ink sacs were separated from *Sepia esculenta* and stored at −70 °C until use. Thawed frozen squid ink at 4 °C and suspended in phosphate buffered solution (0.01M PBS, pH 7.4). The suspension was ultrasonicated and continuously stirred at 4 °C for more than 8 h, and then centrifuged at 4 °C, 8000 rpm to obtain supernatant. Papain (1.5 ‰) was used to hydrolyze the supernatant at 50 °C for 90 min, and it was then heated in boiling water to denature the protease. The protein in the treated supernatant was then removed by the Sevag method. Finally, the polysaccharide was precipitated by mixing the aqueous phase with ethanol of four times the volume, SIP was harvested by freeze drying process.

### 4.2. Preparation of WCPP

Fresh Chinese water chestnuts were purchased from an orchard located in Hezhou City of Guangxi Zhuang autonomous region, China). The peel part was separated and cleaned with distilled water, then dried in an air-forced drier at 40 °C for 24 h, and then pulverized into powder. CWCP powder was mixed with 0.01M PBS at a solid-liquid ratio of 1:30, which was treated with ultrasonic cell crusher, 250 W for 5 min and then kept stirring for more than 8 h at 4 °C. Following centrifugation (8000 rpm) for 15 min, the supernatant was hydrolyzed with papain (1.5 ‰) at 50 °C for 90 min. The enzyme was inactivated in boiling water bath, and proteins in the extract were precipitated with Sevag method. WCPP was obtained by freeze-drying after the polysaccharides in the extract were precipitated with four times the volume of ethanol.

### 4.3. Preincubation of S. cerevisiae

*S. cerevisiae* (ATCC^®^ 204508™) strain was resuscitated through inoculating in solid yeast extract peptone dextrose (YPD) medium (Qingdao Haibo Biotechnology Co., Ltd., China) and cultured for 48 h at 30 °C. Monoclonal colonies were selected from the medium, inoculated in liquid YPD medium and placed in the constant temperature incubator shaker for overnight culture at 30 °C, 150 rpm. Cells were then harvested by centrifugation (3 min, 3000 rpm), resuspended with PBS, and the number of cells was counted.

### 4.4. Model of AA Induced Cell Damage

Cell (initial cell density of 5 × 10^5^ cells /mL) were cultured shakily in liquid YPD medium containing different concentrations (0 mM, 10 mM, 20 mM, 40 mM, 80 mM, 160 mM) of AA (Beijing Dingguo Changsheng Biotechnology Co., Ltd., China) at 30 °C for 20 h. The turbidity of the medium was monitored at 630 nm every two hours with the microplate reader, and the growth curves were plotted by nonlinear regression. The growth curve parameters were subjected to determine the state of cell proliferation treated with AA and the optimal dosage of AA that was used in the following experiment.

For spot assay, when yeast proliferated in liquid YPD with or without 80 mM AA for 6 h, the culture was diluted by gradient (10^7^,10^6^,10^5^,10^4^ cells/mL), and the diluted culture was inoculated with 3 μL into solid YPD medium, and incubated at 30 °C for 48 h.

### 4.5. Effect of Polysaccharides on AA Induced Oxidative Damage in Yeast Model

Experiment was scheduled four groups of yeast that were cultured in different medium, vehicle group (normal medium), AA group (80 mM of AA in normal medium), polysaccharide groups (different concentrations (0.125, 0.25, 0.5, 1, 2 mg/mL) of SIP, WCPP or in normal medium, FL (purchased from XI’AN Bai Chuan Biotech CO., Ltd., China)) and combination group (80 mM of AA and different (0.125, 0.25, 0.5, 1, 2 mg/mL) of SIP, FL or WCPP in normal medium). After inoculation (initial cell density of 5 × 10^5^ cells/mL), cells were cultured, growth curves were plotted by nonlinear fitting and the spot assay was carried out. The kinetic parameters of cells treated with different reagents were calculated, and the ameliorating effect of polysaccharides on the oxidative damage induced by AA was evaluated. Furthermore, the polysaccharide concentration which antagonized oxidative stress effectively was used in next step of the experiment.

### 4.6. Determination of Antioxidant Biomarkers in Yeast Model

Yeasts cultured to different time points were harvested via centrifugation at 4 °C, 3000 rpm for 3 min. Cell precipitate was washed twice and resuspended with ice-cold PBS. The yeast was broken with the ultrasonic cell breaker (Ningbo Shuangjia Instrument Co., Ltd., China), 200W power for 3 s, five times of repeat at 10 s interval, the sample was placed in the ice box at all time. After broken, the cell extracts were clarified by centrifugation (10 min, 8000 rpm at 4 °C) and were used to quantify the protein content by micro BCA protein assay kit (Sangon Biotech Co., Ltd., China). Furthermore, the MDA level was assessed with enzyme-linked immunoassay kit (Jiangsu yutong biotechnology Co., Ltd., China). GSH level and SOD activity were detected by assay kits purchased from Nanjing jiancheng Bioengineering Institute (Nanjing, China).

### 4.7. Kinetic Parameters Calculation and Statistical Analysis

Gompertz models were chosen to describe the state of yeast proliferation in different groups, and the growth curve equation obtained by nonlinear fitting is as follows:

(1) $$Y=Y_{m}\times {\left(Y_{0}/Y_{m}\right)}^{\exp\left(-K\times t\right)}$$

where Y is the OD_630_ of cell at time t (h), Y_m_ is the OD_630_ of cell growth to a stable period, Y_0_ is the starting OD_630_, and the 1/K is the inflection point that determines the lag time (h). Moreover, the doubling time (Dt) of cell proliferation was also an indicator which obtain from transform OD_630_ values using Y = log(Y) and exponential growth with log model of GraphPad Prism 8. To assess the cytotoxic effects of different concentrations of AA, the area under the growth curve (AUC) as obtained by integration and the inhibition rate of AA on cells were calculated by the following formula:

(2) $$\mathrm{Inhibition}\;\mathrm{rate}\left(\%\right)=\left({\mathrm{AUC}}_{\mathrm{vehicla}}-{\mathrm{AUC}}_{\mathrm{AA}}\right)/{\mathrm{AUC}}_{\mathrm{vehicla}}\times 100$$

The statistical analysis was performed using SPSS 19.0 and all data were present as mean ± SD from three independent experiments. Among them, OD_630_ between groups at each time point and the kinetic parameters of cell proliferation in different treatment groups were analyzed for the statistical significance of differences by *t*-test. As for SOD activity, GSH level and MDA level, the statistical significance of differences at different time points and in different treatment groups was analyzed by a two-way repeated measures analysis of variance (RM-ANOVA) with LSD test.

## Figures and Tables

**Table 1:** The inhibition rate of AA at concentrations of 10, 20, 40, 80 and 160 mM to yeasts.

Groups	Vehicle	AA					IC50 (mM)
Inhibition rate (%)	0	Concentration (mM)					92.64 ± 3.30
		10	20	40	80	160	
		0.41 ± 2.6	4.73 ± 3.07	12.65 ± 2.58 *	36.83 ± 1.52 *	80.91 ± 0.17 *	

* Indicated *p* < 0.05 vs. vehicle group.

**Table 2:** The doubling time (Dt) of yeasts under AA at concentrations of 10, 20, 40, 80 and 160 mM, or under polysaccharides at concentrations of 0.125, 0.25, 0.5, 1 and 2 mg/mL.

Groups	Vehicle	AA							Polysaccharide				
			Concentration (mM)						Concentration (mg/mL)				
			10	20	40	80	160		0.125	0.25	0.5	1	2
								SIP	4.66 ± 0.02 *	4.65 ± 0.01 *	4.78 ± 0.05 *	4.88 ± 0.02	4.99 ± 0.04
Dt (h)	4.91 ± 0.02		4.95 ± 0.05	5.12 ± 0.08 *	5.43 ± 0.06 *	7.02 ± 0.08 *	12.26 ± 0.20 *	FL	4.96 ± 0.08	4.93 ± 0.09	4.96 ± 0.05	4.98 ± 0.05	5.00 ± 0.05
								WCPP	4.68 ± 0.01 *	4.66 ± 0.02 *	4.71 ± 0.05 *	4.81± 0.02 *	4.90 ± 0.03

* Indicated *p* < 0.05 vs. vehicle group.

**Table 3:** The Maximum cell proliferation density (Ym), lag time (1/K) and coefficient of determination for growth curves under AA at concentrations of 10,20,40,80 and 160 mM, or under polysaccharides at concentrations of 0.125, 0.25, 0.5, 1 and 2 mg/mL.

Groups	Vehicle	AA						Polysaccharide				
Ym	1.80 ± 0.02	Concentration (mM)						Concentration (mg/mL)				
		10	20	40	80	160		0.125	0.25	0.5	1	2
		1.80 ± 0.02	1.76 ± 0.03	1.66 ± 0.03 *	1.49 ± 0.02 *	0.31 ± 0.01 *	SIP	1.85 ± 0.00 *	1.86 ± 0.01 *	1.88 ± 0.01 *	1.85 ± 0.00 *	1.79 ± 0.01
							FL	1.80 ± 0.01	1.81 ± 0.02	1.81 ± 0.02	1.80 ± 0.01	1.77 ± 0.01
							WCPP	1.87 ± 0.00 *	1.88 ± 0.01 *	1.90 ± 0.01 *	1.86 ± 0.02 *	1.79 ± 0.01
1/K (h)	3.32 ± 0.02	3.36 ± 0.04	3.48 ± 0.03 *	3.65 ± 0.02 *	4.77 ± 0.02 *	5.60 ± 0.15 *	SIP	3.15 ± 0.01 *	3.14 ± 0.01 *	3.27 ± 0.03	3.36 ± 0.02	3.39 ± 0.02 *
							FL	3.36 ± 0.05	3.34 ± 0.05	3.39 ± 0.03	3.38 ± 0.03	3.36 ± 0.03
							WCPP	3.20 ± 0.00 *	3.19 ± 0.01 *	3.26 ± 0.04	3.28 ± 0.04	3.31 ± 0.03
R2	0.9919	0.9936	0.993	0.9888	0.9884	0.9796	SIP	0.9921	0.9928	0.9938	0.9945	0.9921
							FL	0.9907	0.9907	0.9912	0.9926	0.9911
							WCPP	0.9915	0.9916	0.9916	0.9907	0.9916

* indicated *p* < 0.05 vs. vehicle group.

**Table 4:** The doubling time (Dt) of yeasts and the inhibition rate under 80 mM AA or 80 mM AA co-treated with SIP or FL or WCPP administration at concentrations of 0.125, 0.25, 0.5, 1 and 2 mg/mL.

Groups	Vehicle	80 mM AA		Combination				
				Concentration of Polysaccharide (mg/mL)				
				0.125	0.25	0.5	1	2
			SIP + 80 mM AA	6.60 ± 0.05 *	6.75 ± 0.01 *	6.84 ± 0.13 *	7.30 ± 0.05 *	7.82 ± 0.02
Dt (h)	5.23 ± 0.02 *	7.73 ± 0.04	FL + 80 mM AA	7.63 ± 0.20	7.23 ± 0.18 *	7.16 ± 0.15 *	7.41 ± 0.08 *	7.54 ± 0.07 *
			WCPP + 80 mM AA	7.07 ± 0.19 *	7.09 ± 0.09 *	7.23 ± 0.07 *	7.63 ± 0.03 *	7.87 ± 0.06 *
			SIP + 80 mM AA	29.69 ± 0.32 *	31.31 ± 0.46 *	31.91 ± 0.01 *	35.84 ± 0.98 *	40.35 ± 0.17
Inhibition rate (%)	0 *	39.76 ± 0.50	FL + 80 mM AA	38.04 ± 1.15	34.69 ± 1.41 *	34.10 ± 1.12 *	36.13 ± 0.67 *	37.04 ± 0.35 *
			WCPP + 80 mM AA	34.96 ± 1.07 *	34.16 ± 0.86 *	34.41 ± 0.01 *	37.31 ± 0.34 *	39.96 ± 0.25

* Indicated *p* < 0.05 vs. 80 mM AA group.

**Table 5:** The Maximum cell proliferation density (Ym), lag time (1/K) and coefficient of determination for growth curves under 80 mM AA or 80 mM AA co-treated with SIP or FL or WCPP administration at concentrations of 0.125, 0.25, 0.5, 1 and 2 mg/mL.

Groups	Vehicle	80 mM AA				Combination		
				Concentration of Polysaccharide (mg/mL)				
				**0.125**	**0.25**	**0.5**	**1**	**2**
			SIP + 80 mM AA	1.57 ± 0.17 *	1.58 ± 0.00 *	1.60 ± 0.01 *	1.59 ± 0.01 *	1.58 ± 0.01 *
Ym	1.83 ± 0.02 *	1.53 ± 0.00	FL + 80 mM AA	1.60 ± 0.02 *	1.57 ± 0.02	1.56 ± 0.02	1.57 ± 0.02	1.53 ± 0.02
			WCPP + 80 mM AA	1.55 ± 0.03	1.60 ± 0.00 *	1.62 ± 0.02 *	1.61 ± 0.02 *	1.60 ± 0.01 *
			SIP + 80 mM AA	5.17 ± 0.03 *	5.32 ± 0.01 *	5.42 ± 0.09 *	5.70 ± 0.01 *	6.03 ± 0.05 *
1/K (h)	3.91 ± 0.01 *	5.82 ± 0.01	FL + 80 mM AA	5.89 ± 0.08	5.53 ± 0.10 *	5.44 ± 0.05 *	5.61 ± 0.03 *	5.58 ± 0.03 *
			WCPP + 80 mM AA	5.52 ± 0.13 *	5.60 ± 0.03 *	5.66 ± 0.02 *	5.85 ± 0.05	6.04 ± 0.07 *
			SIP + 80 mM AA	0.988	0.9897	0.9907	0.991	0.991
R2	0.9896	0.9898	FL + 80 mM AA	0.9892	0.9886	0.9893	0.9892	0.9885
			WCPP + 80 mM AA	0.9891	0.9876	0.9864	0.9848	0.9861

* Indicated *p* < 0.05 vs. 80 mM AA group.

## Supplementary Materials

The following are available online at https://www.mdpi.com/1660-3397/18/8/395/s1, S1–S2, The OD630 values of different groups of yeasts at each time.
