# Supplementary material for: Preventive Effects of Three Polysaccharides on the Oxidative Stress Induced by Acrylamide in a Saccharomyces cerevisiae Model
Source: Mar Drugs. 2020 Jul 28;18(8):395. doi: 10.3390/md18080395 (PMC7459515; doi:10.3390/md18080395)
Supplement: Supplementary file 1 [file marinedrugs-18-00395-s001.pdf]

Supplementary

Preventive Effects of Three Polysaccharides on the Oxidative Stress Induced by Acrylamide in a *Saccharomyces cerevisiae* Model

Zhen Lin, Yu Zhang, Fangping Li, Xiaohui Tan, Ping Luo and Huazhong Liu*

Table 1

Time (h)	vehicle	AA					SIP					FL					WCPP				
		Concentration of AA (mM)					Concentration of polysaccharides (mg/mL)														
		10	20	40	80	160	0.125	0.25	0.5	1	2	0.125	0.25	0.5	1	2	0.125	0.25	0.5	1	2
2	0.103 ±0.006	0.105 ±0.005	0.101 ±0.012	0.102 ± 0.004	0.088 ±0.003*	0.075 ±0.001*	0.135 ±0.001*	0.138 ±0.002*	0.120 ±0.006*	0.108 ±0.001	0.097 ±0.001	0.104 ±0.006	0.101 ±0.007	0.099 ±0.005	0.098 ±0.005	0.095 ±0.001	0.130 ±0.003*	0.130 ±0.002*	0.119 ±0.005*	0.099 ±0.002	0.113 ±0.001
4	0.259 ±0.003	0.263 ± 0.011	0.246 ±0.011	0.228 ± 0.008*	0.155 ±0.006*	0.109 ±0.001*	0.321 ±0.006*	0.333 ±0.002*	0.280 ±0.012*	0.278 ±0.004*	0.251 ±0.005	0.247 ±0.015	0.262 ±0.018	0.248 ±0.013	0.251 ±0.010	0.249 ±0.009	0.323 ±0.003*	0.328 ±0.004*	0.313 ±0.014*	0.279 ±0.010	0.258 ±0.006
6	0.531 ±0.003	0.547 ± 0.017	0.496 ±0.022	0.424 ±0.016*	0.267 ± 0.003*	0.155 ± 0.002*	0.651 ±0.005*	0.655 ±0.006*	0.594 ±0.015*	0.565 ±0.006*	0.506 ±0.015	0.541 ±0.027	0.541 ±0.034	0.536 ±0.032	0.528 ±0.015	0.520 ±0.013	0.639 ±0.007*	0.644 ±0.008*	0.638 ±0.018*	0.613 ±0.011*	0.561 ±0.007*
8	0.999 ±0.017	0.926 ±0.026*	0.860 ±0.016*	0.719 ± 0.012*	0.421 ±0.012*	0.192 ± 0.003*	1.055 ±0.003*	1.057 ±0.005*	1.025 ±0.018	0.970 ±0.006	0.931 ±0.034	0.936 ±0.032	0.949 ±0.031	0.935 ±0.026*	0.927 ±0.020*	0.921 ±0.023*	1.055 ±0.003*	1.057 ±0.005*	1.025 ±0.018	0.970 ±0.006	0.931 ±0.034
10	1.419 ±0.005	1.374 ± 0.029	1.270 ±0.034*	1.117 ±0.026*	0.629 ±0.006*	0.229 ±0.005*	1.515 ±0.008*	1.514 ±0.008*	1.476 ±0.021*	1.402 ±0.009*	1.406 ±0.013	1.399 ±0.028	1.426 ±0.034	1.426 ±0.029	1.401 ±0.023	1.388 ±0.017	1.508 ±0.005*	1.532 ±0.006*	1.516 ±0.020*	1.500 ±0.006*	1.438 ±0.018
12	1.493 ±0.022	1.551 ±0.024*	1.513 ±0.029	1.444 ±0.026	0.892 ±0.015*	0.255 ± 0.006*	1.602 ±0.003*	1.625 ±0.010*	1.639 ±0.014*	1.593 ±0.005*	1.488 ±0.017	1.548 ±0.014*	1.550 ±0.025*	1.514 ±0.024	1.511 ±0.013	1.519 ±0.030	1.604 ±0.004*	1.616 ±0.012*	1.622 ±0.018*	1.606 ±0.010*	1.533 ±0.004
14	1.581 ±0.018	1.580 ±0.024	1.512 ±0.026*	1.445 ±0.019*	1.068 ±0.004*	0.276 ±0.009*	1.623 ±0.002*	1.642 ±0.012*	1.648 ±0.014*	1.601 ±0.011	1.567 ±0.023	1.545 ±0.006	1.568 ±0.021	1.553 ±0.021	1.567 ±0.009	1.525 ±0.034	1.623 ±0.002*	1.642 ±0.012*	1.648 ±0.014*	1.601 ±0.011	1.567 ±0.023
16	1.637 ±0.034	1.654 ±0.026	1.619 ± 0.027	1.496 ±0.031*	1.285 ± 0.015*	0.295 ± 0.013*	1.729 ±0.005*	1.746 ±0.010*	1.763 ±0.015*	1.718 ±0.004*	1.671 ±0.017	1.647 ±0.013	1.658 ±0.020	1.645 ±0.018	1.659 ±0.010	1.623 ±0.018	1.733 ±0.001*	1.762 ±0.006*	1.758 ±0.013*	1.718 ±0.006	1.701 ±0.002
18	1.760 ±0.014	1.757 ±0.009	1.713 ±0.027	1.559 ± 0.022*	1.291 ±0.017*	0.291 ± 0.005*	1.824 ±0.004*	1.826 ±0.008*	1.818 ±0.015*	1.804 ±0.010*	1.713 ±0.016*	1.717 ±0.026	1.733 ±0.033	1.756 ±0.029	1.723 ±0.017	1.701 ±0.013*	1.856 ±0.005*	1.847 ±0.005*	1.862 ±0.017*	1.836 ±0.008*	1.749 ±0.022
20	1.819 ±0.024	1.773 ±0.020	1.697 ± 0.030*	1.597 ±0.030*	1.287 ±0.019*	0.275 ±0.004*	1.876 ±0.006*	1.877 ±0.008*	1.875 ±0.019*	1.843 ±0.007	1.785 ±0.009	1.834 ±0.024	1.825 ± 0.029	1.824 ±0.023	1.807 ± 0.021	1.773 ±0.008	1.916 ±0.014*	1.926 ±0.018*	1.949 ± 0.014*	1.860 ±0.035	1.752 ±0.023*

Table 1 The OD630 values of different groups of yeasts at each time. * indicated $p<0.05$ vs. corresponding time point at vehicle group

Table 2

Time (h)	vehicle	80mM AA	SIP+80mM AA					FL+80mM AA					WCPP+80mM AA				
			Concentration of polysaccharides (mg/mL)														
			0.125	0.25	0.5	1	2	0.125	0.25	0.5	1	2	0.125	0.25	0.5	1	2
2	0.093 ±0.005*	0.082 ±0.002	0.122 ±0.012 *	0.112 ±0.001*	0.108 ±0.005 *	0.094 ±0.001*	0.090 ±0.001*	0.092 ±0.004 *	0.093 ±0.005 *	0.090 ±0.004*	0.088 ±0.002 *	0.084 ±0.002	0.106 ±0.005*	0.104 ±0.004*	0.100 ±0.003 *	0.088 ±0.001 *	0.087 ±0.002*
4	0.219 ±0.005*	0.130 ±0.001	0.193 ±0.010 *	0.183 ±0.001 *	0.175 ±0.009 *	0.151 ±0.002 *	0.140 ±0.001*	0.151 ±0.006 *	0.153 ±0.007*	0.154 ±0.006*	0.148 ±0.002 *	0.144 ±0.003*	0.169 ±0.008 *	0.168 ±0.006 *	0.162 ±0.006 *	0.144 ±0.001 *	0.136 ±0.002 *
6	0.461 ±0.007*	0.226 ±0.003	0.303 ±0.008 *	0.297 ±0.004 *	0.285 ±0.012 *	0.253 ±0.001 *	0.221 ±0.003	0.222 ±0.009	0.258 ±0.012 *	0.260 ±0.009 *	0.246 ±0.005 *	0.236 ±0.002 *	0.263 ±0.009 *	0.264 ±0.008 *	0.256 ±0.008 *	0.232 ±0.002	0.232 ±0.004
8	0.845 ±0.011*	0.362 ±0.007	0.469 ±0.005 *	0.459 ±0.005 *	0.450 ±0.017 *	0.400 ±0.005 *	0.353 ±0.002	0.374 ±0.017	0.406 ±0.017 *	0.413 ±0.015 *	0.384 ±0.006 *	0.375 ±0.008	0.405 ±0.012 *	0.400 ±0.005 *	0.398 ±0.015 *	0.365 ±0.005	0.346 ±0.008
10	1.335 ±0.004*	0.581 ±0.012	0.676 ±0.005*	0.648 ±0.003 *	0.647 ±0.007 *	0.601 ±0.009 *	0.543 ±0.002*	0.561 ±0.022	0.613 ±0.022	0.633 ±0.020 *	0.599 ±0.012 *	0.591 ±0.010	0.634 ±0.042	0.632 ±0.017 *	0.604 ±0.031	0.573 ±0.020	0.528 ±0.000 *
12	1.478 ±0.009*	0.742 ±0.007	0.912 ±0.008 *	0.880 ±0.005 *	0.862 ±0.011 *	0.807 ±0.012 *	0.742 ±0.009	0.784 ±0.015 *	0.846 ±0.031*	0.858 ±0.018 *	0.827 ±0.013 *	0.839 ±0.012*	0.824 ±0.004 *	0.820 ±0.006 *	0.828 ±0.012*	0.774 ±0.002*	0.731 ±0.019
14	1.540 ±0.021*	0.985 ±0.007	1.158 ±0.009 *	1.117 ±0.004 *	1.109 ±0.014 *	1.044 ±0.006 *	0.961 ±0.009*	1.012 ±0.022	1.091 ±0.030 *	1.105 ±0.022 *	1.068 ±0.016 *	1.051 ±0.015 *	1.038 ±0.012 *	1.056 ±0.004 *	1.067 ±0.022 *	1.026 ±0.003 *	0.992 ±0.012
16	1.609 ±0.019*	1.173 ±0.003	1.303 ±0.006 *	1.289 ±0.005 *	1.270 ±0.011 *	1.217 ±0.004 *	1.147 ±0.007 *	1.205 ±0.021	1.247 ±0.025 *	1.254 ±0.021 *	1.224 ±0.016 *	1.216 ±0.013 *	1.225 ±0.009 *	1.272 ±0.003 *	1.267 ±0.014 *	1.239 ±0.015*	1.176 ±0.014
18	1.702 ±0.005*	1.220 ±0.005	1.335 ±0.005 *	1.321 ±0.007 *	1.337 ±0.016 *	1.301 ±0.015 *	1.236 ±0.011	1.249 ±0.025	1.270 ±0.027 *	1.271 ±0.020 *	1.262 ±0.021 *	1.223 ±0.017	1.298 ±0.004 *	1.318 ±0.001 *	1.331 ±0.015 *	1.295 ±0.003 *	1.251 ±0.013 *
20	1.811 ±0.016*	1.226 ±0.007	1.339 ±0.022 *	1.342 ±0.007 *	1.344 ±0.006 *	1.293 ±0.010 *	1.242 ±0.003	1.282 ±0.029	1.306 ±0.031 *	1.309 ±0.036 *	1.281 ±0.020 *	1.263 ±0.016 *	1.279 ±0.008 *	1.301 ±0.005 *	1.294 ±0.016 *	1.251 ±0.013	1.229 ±0.009

Table 2 The OD630 values of different groups of yeasts at each time. * indicated $p<0.05$ vs. corresponding time point at vehicle group
